# Prevalence of Hepatitis B Virus, Hepatitis C Virus, and HIV Infections in Hemodialysis Patients at Kano Kidney Center

**DOI:** 10.7759/cureus.41769

**Published:** 2023-07-12

**Authors:** Ridha H Alkhalifah, Mousa J Alhaddad, Ali T Alhashem, Hussain Alwesaibi, Abdullah A AlKhalaf, Abdullah Albin Saad, Mohammed Almattar, Makarem A Alkhalaf, Habib Alramadhan, Mohammad Albaggal

**Affiliations:** 1 Department of Internal Medicine, Dammam Medical Complex, Dammam, SAU; 2 College of Clinical Pharmacy, Imam Abdulrahman Bin Faisal University, Dammam, SAU; 3 Nephrology, Kano Kidney Center, Dammam Medical Complex, Dammam, SAU

**Keywords:** saudi arabia, human immunedeficiecy virus (hiv) infection, hepatitis c virus (hcv), hepatitis b virus (hbv), maintenance hemodialysis, end stage renal disease (esrd)

## Abstract

Background

Hepatitis B virus (HBV), hepatitis C virus (HCV), and human immunodeficiency virus (HIV) infections are more prevalent in hemodialysis patients compared to the general population. The objective of this study was to evaluate the prevalence of HBV, HCV, and HIV infections in hemodialysis patients dialyzing regularly at Kano Kidney Center (KKC) in the Eastern Health Cluster of Saudi Arabia in 2022.

Methods

This retrospective study included all hemodialysis patients who were dialyzed regularly at KKC during 2022. Their electronic medical records were reviewed for the results of HBV, HCV, and HIV along with the patient's demographics, comorbid conditions, and dialysis history. The study was approved and monitored by the Institutional Review Board of Dammam Medical Complex.

Results

A total of 239 regular hemodialysis patients were included, consisting of 142 males and 97 females (59.41% and 40.59%, respectively), with a mean age of 52.71±15.83 years. Most of the patients were Saudis (156 patients, 65.27%) with the non-Saudi patients being composed mostly of Arabian patients. Nine patients (3.77%) tested positive for hepatitis B surface antigen (HBsAg), the serologic hallmark of HBV infection. Two patients (0.84%) had resolved HBV infections as evidenced by positive hepatitis B core antibody (anti-HBc) and hepatitis B surface antibody (anti-HBs). However, the majority (226 patients, 94.56%) were never tested for anti-HBc. Anti-HBs, which can imply long-term immunity against HBV from prior immunizations or infections, were positive in 165 patients (69.04%). A protective anti-HBs level of ≥ 10 IU/L was detected in 158 patients (66.11%) including 104 patients (43.51%) having ≥ 100 IU/L. Eighteen patients (7.53%) had reactive HCV antibodies. Four patients (1.67%) had chronic HCV infection as they had detectable HCV RNA. The remaining 14 patients (5.86%) cleared HCV either spontaneously (seven patients, 2.93%) or by medications (seven patients, 2.93%). HIV screening tests were negative in all 239 patients (100%).

HBsAg-positive patients did not have any statistically significant differences from HBsAg-negative patients. On the other hand, the patients who were positive for HCV antibodies were older than the patients who were negative for HCV antibodies (60.66 vs 52.05 years on average, p-value <0.05). They also contained a statistically larger proportion of non-Saudi patients than the patients with no evidence of prior infections (61.11% vs 32.13%, p-value <0.05).

Conclusions

The study found that the prevalence of HBV and HCV infections among hemodialysis patients in KKC at 3.77% and 1.67%, respectively, is higher than that reported in the general population in Saudi Arabia, with non-Saudis having a higher prevalence rate of HCV infection than Saudis. However, the current prevalence rate is lower compared to the previous studies that were conducted in Saudi Arabia in the first decade of the 21st century, and there were no cases of HIV infections. Nevertheless, a significant proportion of patients had unprotective or negative anti-HBs antibody titers, indicating the need for strict vaccination protocols and monitoring of antibody titers to ensure optimal protection.

## Introduction

Hepatitis B virus (HBV), hepatitis C virus (HCV), and human immunodeficiency virus (HIV) infections are more prevalent in hemodialysis patients compared to the overall prevalence in the community. In Middle Eastern countries, the prevalence of HBV infection among blood donors was 1.62% [1], but its prevalence among hemodialysis patients was estimated at 4.4% [2]. For HCV, the overall prevalence was found to be 4.34% [3], which is much lower than the reported prevalence of 25.3% among hemodialysis Middle Eastern patients [4]. Although the prevalence of HIV of 0.1% in the Middle East is among the lowest rates globally [5], multiple outbreaks of HIV transmission in dialysis centers were reported in the region specifically in Saudi Arabia (2011) [6] and Egypt (1993) [7].

Multiple reasons can explain the association between HBV, HCV, and HIV infections and hemodialysis. Hemodialysis patients are at an increased risk of contracting these infections due to their exposure to contaminated medical equipment used during the hemodialysis process [6-9]. In addition, many hemodialysis patients require blood transfusions for their anemia of chronic kidney disease. If infected, these transfusions can lead to infections. Although this mode of transmission is negligible nowadays, it was more prominent in the past as evidenced by the reported association between HCV seropositivity in hemodialysis patients with the higher numbers of transfused blood units [10,11]. Moreover, patients with chronic kidney disease have a weakened immune system, which can make them less capable of clearing HBV and HCV infections [12-14]. Furthermore, hemodialysis patients have a suboptimal immune response to HBV vaccine compared with the non-dialysis population [14-16]. They were also rarely offered treatment for HCV infections in the past due to the concerns about the efficacy and risks of interferon and ribavirin therapy in this population with a continued trend of undertreatment even after the introduction of new, improved antiviral agents [17,18].

HBV and HCV infections can lead to hepatocellular carcinoma (HCC), and HCC patients on hemodialysis are at greater risk of death than HCC patients not on dialysis [19]. In addition to liver disease-related mortality, these infections in hemodialysis patients are linked to an increased risk of cardiovascular disease-related mortality and all-cause mortality [20-22]. Moreover, HCV-seropositive patients have decreased accessibility to kidney transplantation programs despite having a substantial survival benefit if transplanted [23].

The objective of this study was to evaluate the prevalence of HBV, HCV, and HIV infections in hemodialysis patients dialyzing regularly in Kano Kidney Center (KKC) in the Eastern Health Cluster of Saudi Arabia in 2022.

## Materials and methods

The study was a retrospective chart review study. It included all the hemodialysis patients who came for dialysis regularly to KKC in 2022. Their electronic medical records were reviewed for their demographic data (e.g., age, gender, and nationality), comorbid conditions (e.g., diabetes mellitus and hypertension), and personal dialysis history (e.g., age at the start of hemodialysis, dialysis route and any prior history of kidney transplantation or peritoneal dialysis). The records were also reviewed for the results of HBV, HCV, and HIV. The study was approved and monitored by the Institutional Review Board (IRB) of Dammam Medical Complex on March 20, 2023 (approval no: IM-07).

Chronic HBV infection is defined by the persistence of hepatitis B surface antigens (HBsAg) for more than six months as per the American Association of the Study of Liver Diseases (AASLD) guidelines. Patients with positive hepatitis B surface antibodies (anti-HBs) and hepatitis B core antibodies (anti-HBc) were considered to have resolved HBV infections. An anti-HBs level of 10 IU/L or more was used to define immunity against HBV [24,25]. In keeping with the recommendations of the joint panel from the AASLD and the Infectious Diseases Society of America about HCV testing, chronic HCV infection is defined by the presence of reactive anti-HCV antibodies and detectable HCV RNA. The absence of detectable HCV RNA in the setting of positive anti-HCV antibodies was used to indicate resolved infections. Patients with resolved HCV infections were further divided into those who spontaneously cleared the virus and those who were successively treated.

The prevalence of HBV was calculated as follows: (the number of HBsAg-positive patients × 100)/(the number of all patients). The prevalence of HCV was calculated as follows: (the number of patients with reactive anti-HCV antibodies who had detectable HCV RNA × 100)/(the number of all patients).

The patients with chronic HBV infection (i.e., HBsAg-positive patients) were compared with the HBsAg-negative patients looking for any association between HBV infection and the patient's demographics, comorbid conditions, and personal dialysis data. Similarly, patients with prior or active HCV infection (i.e., patients with positive HCV antibodies) were compared with the patients with no history of HCV infection.

The data were analysed using the Python programming language version 3.7.6 (Python Software Foundation, Wilmington, Delaware, USA) with the use of the SciPy library 1.4.1 (Enthought, Inc., Austin, Texas, USA), and Statsmodels module (v0.11.1, Python package). Descriptive statistics (i.e., mean, standard deviation, count, and percentage) were calculated as necessary. Categorical variables were compared with the chi-square test, and continuous variables were compared with the two-sample t-test. A p-value of less than 0.05 was assumed to indicate statistical significance.

## Results

A total of 239 hemodialysis patients were included. The patients consisted of 142 males and 97 females (59.41% and 40.59%, respectively), with a male-to-female ratio of 1.46. Most of the patients were Saudis (156 patients, 65.27%) with the non-Saudi patients being composed mostly of Arabian patients. The mean ± standard deviation for the patients' age was 52.71±15.83 years. Two hundred sixteen patients (90.38%) were hypertensive. One hundred thirty-three patients (55.65%) were diabetic. The patients' demographics are shown in Table 1.

**Table 1:** Patient Demographics (n = 239)

Characteristics		n (%)
Age (Mean ± SD, years)		52.71±15.83
Gender	Male	142 (59.41%)
	Female	97 (40.59%)
Nationality	Saudi	156 (65.27%)
	Non-Saudi	83 (34.73%)
Hypertension		216 (90.38%)
Diabetes Mellitus	Type 1	14 (5.86%)
	Type 2	119 (49.79%)
	Non-diabetic	106 (44.35%)

The end-stage renal disease (ESRD) etiology was attributed in more than half of the patients (120 patients, 50.21%) to diabetes mellitus. Thirteen patients (5.44%) had a prior history of kidney transplantation. Ten patients (4.18%) were previously on peritoneal dialysis. The mean ± standard deviation for the patient's age at the start of hemodialysis and duration of hemodialysis was 50.43±16.27 years and 1.99±1.83 years, respectively. Most of the patients (147 patients, 61.51%) were dialyzed through dialysis lines. The remaining 92 patients (38.49%) were dialyzed through arteriovenous fistulas. Two hundred twenty patients (92.05%) had three scheduled hemodialysis sessions per week. Twenty-two patients (9.21%) occasionally went to other centers for dialysis. The dialysis history information of patients is shown in Table 2.

**Table 2:** Dialysis History of Patients (n = 239)

Characteristics		n (%)
ESRD Etiology	Diabetes Mellitus	120 (50.21%)
	Hypertension	56 (23.43%)
	Other Causes	28 (11.72%)
	Unknown	35 (14.64%)
Prior History of Kidney Transplantation		13 (5.44%)
Prior History of Peritoneal Dialysis		10 (4.18%)
Age at Start of Hemodialysis (Mean ± SD, years)		50.43±16.27
Duration of Hemodialysis (Mean ± SD, years)		1.99±1.83
Hemodialysis Route	Dialysis Line	147 (61.51%)
	Arteriovenous Fistula	92 (38.49%)
Hemodialysis Sessions per Week	2	18 (7.53%)
	3	220 (92.05%)
	4	1 (0.42%)
Dialyzing in Another Facility		22 (9.21%)

Nine patients (3.77%) tested positive for HBsAg, the serologic hallmark of HBV infection. Anti-HBs, which can imply long-term immunity against HBV from prior immunizations or infections, were positive in 165 patients (69.04%). An anti-HBs level of ≥ 10 IU/L was detected in 158 patients (66.11%) including 104 patients (43.51%) having ≥ 100 IU/L. The coexistence of HBsAg and anti-HBs was not found in any patient. Six patients (2.51%) had documented anti-HBc, which appears during HBV infection and persists even after recovery. However, the majority (226 patients, 94.56%) were never tested for anti-HBc.

**Figure 1:**
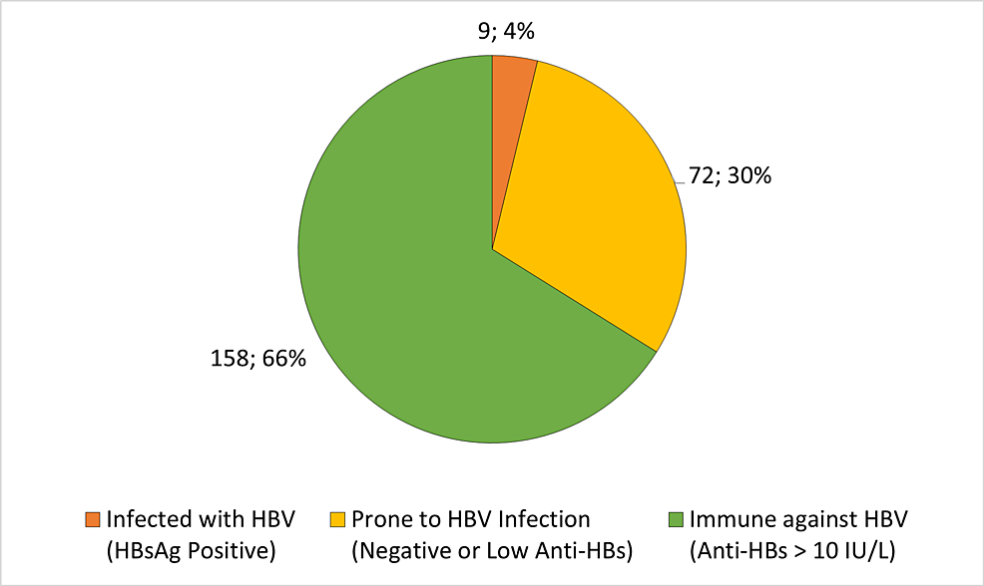
HBV Statuses in 239 ESRD Hemodialysis Patients HBV: hepatitis B virus, HBsAg: hepatitis B surface antigen, Anti-HBs: hepatitis B surface antibody; ESRD: end stage renal disease.

All nine patients (3.77%) with positive HBsAg had a chronic HBV infection with a median viral load of 86 international units/mL and an interquartile range from 19 to 361 international units/mL. Four of them were also positive for anti-HBc. Two patients (0.84%) had resolved HBV infections as evidenced by positive anti-HBc and anti-HBs. The HBV statuses are shown in Figure 1.

**Figure 2:**
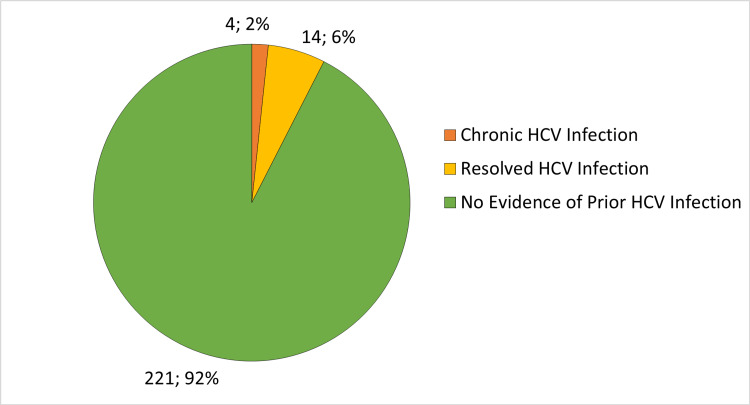
Outcomes of HCV Infections in 239 ESRD Hemodialysis Patients HCV: hepatitis C virus; ESRD: end stage renal disease.

Eighteen patients (7.53%) had reactive HCV antibodies. Four patients (1.67%) had chronic HCV infection as they had detectable HCV RNA with a median initial viral load of 824809 international units/mL and an interquartile range from 505833 to 1256190.75 international units/mL. The remaining 14 patients (5.86%) cleared HCV either spontaneously (seven patients, 2.93%) or by medications (seven patients, 2.93%). The outcomes of HCV infections are shown in Figure 2.

HIV screening tests were negative in all the 239 patients (100%). Results of HBV, HCV, and HIV investigations are shown in Table 3.

**Table 3:** HBV, HCV, and HIV Investigations of Patients (n = 239) HBV: hepatitis B virus; HCV: hepatitis C virus; HIV: human immunodeficiency virus.

Characteristics		n (%)
Hepatitis B Surface Antigen (HBsAg)	Negative	230 (96.23%)
	Positive	9 (3.77%)
Hepatitis B Surface Antibody (anti-HBs)	Negative	73 (30.54%)
	Positive	165 (69.04%)
	Not done	1 (0.42%)
Hepatitis B Core Antibody (anti-HBc)	Negative	7 (2.93%)
	Positive	6 (2.51%)
	Not done	226 (94.56%)
HCV Antibody	Negative	221 (92.47%)
	Positive	18 (7.53%)
HIV Screen (Antigen and Antibody)	Negative	239 (100.0%)

There were no statistically significant differences between HBsAg-positive and HBsAg-negative patients. On the other hand, the 18 patients who had HCV infections currently or previously were older than the patients with no evidence of prior HCV infection (60.66 vs 52.05 years on average, p-value <0.05). They also contained a statistically larger proportion of non-Saudi patients than the patients with no evidence of prior infections (61.11% vs 32.13%, p-value <0.05). Three patients were Yemeni. Two patients each were from Egypt, Sudan, and Pakistan. The remaining two non-Saudi patients were Palestinian and Indian. The detailed comparisons between the patients with positive and negative HBsAg and HCV antibodies are shown in Tables 4-5, respectively. 

**Table 4:** Comparison between Patients with Chronic HBV Infection and Patients with no Current HBV Infection (n = 239) HBV: hepatitis B virus; HBsAg: hepatitis B surface antigen.

Characteristics	Negative HBsAg (n = 230)	Positive HBsAg (n = 9)	P value
Age, mean ± SD, years	52.74±15.99	52.05±11.85	0.8987
Male sex, count (%)	135 (58.7%)	7 (77.78%)	0.4251
Non-Saudi, count (%)	78 (33.91%)	4 (44.44%)	0.768
Hypertension, count (%)	207 (90.0%)	9 (100.0%)	0.6732
Diabetes Mellitus, count (%)	127 (55.22%)	6 (66.67%)	0.7367
Prior History of Peritoneal Dialysis, count (%)	9 (3.91%)	1 (11.11%)	0.8341
Prior History of Kidney Transplantation, count (%)	12 (5.22%)	1 (11.11%)	0.9875
Age at Start of Hemodialysis, mean ± SD, years	50.51±16.29	47.27±17.56	0.6954
Duration of Hemodialysis, mean ± SD, years	1.99±1.85	2.21±1.21	0.8108
Dialysis Sessions per Week, mean ± SD	2.93±0.28	3.0±0.0	0.4274
Arteriovenous Fistula, count (%)	88 (38.26%)	4 (44.44%)	0.9802
Dialysis at Another Facility, count (%)	22 (9.57%)	0 (0.0%)	0.6995

**Table 5:** Comparison between Patients with Prior or Active HCV Infection and Patients with no Evidence of Previous HCV Infection (n = 239) * A p-value of less than 0.05 was used to indicate statistical significance. HCV: hepatitis C virus.

Characteristics	Positive HCV Antibodies (n = 18)	Negative HCV Antibodies (n = 221)	P value
Age, mean ± SD, years	60.66±11.47	52.06±15.98	0.0265*
Male sex, count (%)	8 (44.44%)	134 (60.63%)	0.2733
Non-Saudi, count (%)	11 (61.11%)	71 (32.13%)	0.0256*
Hypertension, count (%)	15 (83.33%)	201 (90.95%)	0.5234
Diabetes Mellitus, count (%)	10 (55.56%)	123 (55.66%)	0.8115
Prior History of Peritoneal Dialysis, count (%)	0 (0.0%)	10 (4.52%)	0.7566
Prior History of Kidney Transplantation, count (%)	2 (11.11%)	11 (4.98%)	0.5734
Age at Start of Hemodialysis, mean ± SD, years	60.06±11.33	50.01±16.35	0.1393
Duration of Hemodialysis, mean ± SD, years	3.04±1.35	1.95±1.84	0.1519
Dialysis Sessions per Week, mean ± SD	3.0±0.34	2.92±0.27	0.2519
Arteriovenous Fistula, count (%)	9 (50.0%)	83 (37.56%)	0.4287
Dialysis at Another Facility, count (%)	3 (16.67%)	19 (8.6%)	0.4747

## Discussion

HBV infection in hemodialysis patients

In this study, the prevalence of HBV infection in hemodialysis patients was 3.8% which is higher than the estimated HBV prevalence in Saudi Arabia (2019) at 1.3% [26] and its reported prevalence of 0.3% among blood donors in eastern Saudi Arabia between 2011 and 2015 [27]. However, it is comparable to the finding of a study done in a dialysis unit in Riyadh (the capital and largest city of central Saudi Arabia) which showed a prevalence of 4.1% [28]. As a comparison, the prevalence of HBV infection among hemodialysis patients is 1.6% in Lebanon (2012) [29], 3.8% in Palestine (2014) [30], 4% in Iran (2018) [31], 5.9% in Jordan (2006) [32], 6% in Morocco (2009) [33], 6.3% in Egypt (2022) [34] and 67.8% in Pakistan (2017) [35]. Fortunately, the current prevalence is markedly lower than the previously reported 11.8% prevalence of HBV infection in hemodialysis patients in the eastern region in a study published in 2004 [36]. This decline in HBV prevalence among hemodialysis patients can reflect a better adherence to infection control practices along with an increased HBV vaccination coverage, an improved HBV screening in dialysis patients and blood donors, and a reduced need for blood transfusions with the increasing use of erythropoiesis-stimulating agents.

Only 66% of the patients were considered immune against HBV by having anti-HBs ≥ 10 IU/L. This is comparable to a response rate of 70.3% following a program of four doses of 40 μg HBV vaccine that was evaluated in another Saudi study [37]. The weak immune response to vaccines in ESRD patients is not limited to the HBV vaccine but is also evident across many other types of vaccines (e.g., coronavirus disease 2019 (COVID-19) and influenza) [38-40]. This impairment of HBV vaccine efficacy in ESRD patients is not different between peritoneal dialysis patients and hemodialysis patients [41]. Many factors were found to affect the HBV vaccine efficacy in hemodialysis patients. The factors that were associated with decreased response to HBV vaccine include old age [37,42], diabetes [42,43], and protein catabolic rate (PCR) [44]. Patients with excellent immune response (anti-HBs ≥ 100 IU/L) at six weeks after completion of the vaccination series have a greater chance of persistent immunity at one year [37]. This fact is also more evident if anti-HBs levels are more than 1000 IU/L [43].

It is recommended that all hemodialysis patients should be screened and vaccinated for HBV which has been found to decrease the rate of infection among this population of patients. Repeating the vaccination series is recommended for patients who do not achieve a protective anti-HBs titer [45]. Poor responders (anti-HBs between 10 and 100 IU/L) can be susceptible to HBV infection over time and, therefore, should be offered a booster vaccination dose if anti-HBs drop below 10 IU/L [43].

HCV infection in hemodialysis patients

In this study, eighteen patients (7.5%) had reactive HCV antibodies but only four patients (1.7%) had chronic HCV infection based on detectable HCV RNA. This prevalence is remarkably lower than it had been in 2006 when it was 29% [46]. Furthermore, in an analysis of 39 studies that evaluated HCV infections in hemodialysis patients between 1991 and 2009, HCV prevalence ranged between 18.9% and 78.2% with a pooled HCV prevalence of 46.9% [47]. Thus, this is the first demonstration of a fall in HCV prevalence in a Saudi dialysis center to a rate approximating the overall HCV seroprevalence in Saudi Arabia of 1.1% [48]. This drop is secondary to the application of multiple preventive measures at the center including separation of HCV-positive from HCV-seronegative patients on different floors with different machines, repetitive teaching of dialysis staff about infection control policies, and reinforcement of disinfection protocols after each dialysis session [46]. 

Direct-acting antiviral (DAA) therapies for HCV are highly effective with a cure rate above 90% in hemodialysis patients [49-52] with many listed available options in the Kidney Disease: Improving Global Outcomes (KDIGO) 2022 Clinical Practice Guideline for the Prevention, Diagnosis, Evaluation, and Treatment of Hepatitis C in Chronic Kidney Disease [53]. Seven patients with chronic HCV infection in this cohort were treated and had sustained virological response, highlighting the efficacy and importance of DAA therapies for HCV. Unfortunately, only two patients out of the four patients with chronic HCV infection were actively receiving DAA therapies, and the other two were not referred to a gastroenterology clinic for evaluation and possible initiation of DAA therapies. It is recommended to follow the KDIGO guidelines that recommend that all patients with chronic kidney disease, patients on hemodialysis, and kidney transplant recipients with HCV should be evaluated for DAA therapies [53].

HIV infection in hemodialysis patients

The prevalence of HIV in Saudi Arabia is very low. Among 375,218 potential blood donors in 2020, only 0.0018% were confirmed to have HIV [54]. The lowest prevalence of HIV was reported in the eastern region of Saudi Arabia [54]. Unexpectedly, there were no patients with positive HIV screening in this study which was conducted at a dialysis center in the Eastern Province. A zero prevalence of HIV among hemodialysis patients was also reported in a dialysis center in Riyadh which is located in the central region of Saudi Arabia [28]. In another center in Riyadh, three patients acquired HIV after reaching the ESRD stage [55]. An outbreak of HIV transmission occurred in 2012 in Jizan, a city in the southern region of Saudi Arabia, where two patients at a hemodialysis unit had become HIV positive [6]. Sharing injections between patients from a multidose heparin vial, using inadequately disinfected hemodialysis equipment, and obtaining vascular access by blood-contaminated gloves were the causes of this transmission. Corrective actions were performed with an emphasis on infection control practices at a national level [6]. No further outbreaks of HIV transmissions in hemodialysis units were reported in Saudi Arabia since then.

Study limitations

The study had several limitations. Firstly, the study was retrospective in nature which can lead to potential biases due to missing information and inaccurate documentation. Secondly, the study was conducted at a single center which may limit the generalizability of the results. The rate of HBV and HCV infections can vary between different dialysis institutions due to differences in patient populations and infection control practices. Moreover, some of the included patients had outdated screening results, as their results were dated one year prior to the study. Thus, it is possible that their infection status may have changed later. Some patients refused to repeat screening due to financial constraints, but new rules were implemented to mandate repeating the virology assessment in a timely manner. Additionally, the study only reported the point prevalence of HBV, HCV, and HIV in hemodialysis patients, and did not examine the seroconversion rates. Therefore, it did not provide information on the incidence of new infections. Furthermore, the number of patients with resolved HBV infection in the study population was underestimated as only 13 patients were tested for anti-HBc. Another limitation of the study is that it did not address false-negative HCV antibodies. Hemodialysis patients are known to have a high false-negative rate of anti-HCV antibodies, despite having detectable HCV RNA [56]. For example, a study conducted in Egypt reported a false-negative rate of 17.9% among 72 hemodialysis patients with negative anti-HCV antibodies [57]. Finally, the study did not address occult HBV infection which is defined as the presence of detectable HBV DNA in patients who are negative for HBsAg [58]. A study conducted in Egypt and another one in Iran reported a rate of 4% of occult HBV infection among 145 and 118 HBsAg-negative hemodialysis patients, respectively [59,60]. 

Future studies should aim to overcome these limitations to provide a more accurate picture of the prevalence and incidence of HBV, HCV, and HIV infections in hemodialysis patients in Saudi Arabia and elsewhere.

## Conclusions

The study found that the prevalence of HBV and HCV infections among hemodialysis patients in KKC at 3.77% and 1.67%, respectively, is higher than that reported in the general population in Saudi Arabia, with non-Saudis having a higher prevalence rate of HCV infection than Saudis. However, the current prevalence rate is lower compared to previous studies in the first decade of the 21st century, and there were no cases of HIV infections. Nevertheless, a significant proportion of patients had unprotective or negative anti-HBs antibody titers, indicating the need for strict vaccination protocols and monitoring of antibody titers to ensure optimal protection.
